# A longitudinal analysis of the prevalence of restrictive interventions involving women with mental health conditions, learning disabilities or autism in mental health services in England

**DOI:** 10.3389/fpsyt.2026.1787826

**Published:** 2026-04-14

**Authors:** Kathryn Fradley, Alina Haines-Delmont

**Affiliations:** School of Nursing and Public Health, Faculty of Health and Education, Manchester Metropolitan University, Manchester, England, United Kingdom

**Keywords:** autism, England, learning disability, longitudinal trends, mental health services, restrictive interventions, women

## Abstract

**Introduction:**

Restrictive interventions, including physical restraint, seclusion, chemical restraint, and segregation, continue to be used within mental health services, despite sustained policy efforts to promote least-restrictive and trauma-informed care. However, little is known about national trends affecting women, for whom restrictive interventions often carry heightened risks of re-traumatisation and stigma.

**Methods:**

We conducted a longitudinal secondary analysis of publicly available administrative data from the Mental Health Bulletin covering NHS-funded mental health services in England between 2017 and 2025. Annual counts of restrictive interventions involving women were examined relative to the number of women detained under the Mental Health Act to estimate annual rates per 1,000 detained. Regression modelling was used to assess temporal trends overall, by age group and type of restrictive intervention, and interrupted time-series analyses to examine changes following implementation of the Mental Health Units (Use of Force) Act 2018 (“Seni’s Law”). Trends were also examined alongside available national data on restrictive interventions involving men.

**Results:**

Rates of restrictive interventions involving women increased by approximately 12 percent per year over the study period, with no evidence of a reduction following the introduction of Seni’s Law. Increases were most pronounced for chemical restraint, seclusion, and segregation, while physical and mechanical restraint remained stable. Restrictive interventions declined among women under 18 but increased consistently across all adult age groups, indicating a widening age-related divergence. Although overall trends broadly mirrored those observed among men, the types of restrictive interventions used and their potential impact may differ, highlighting gendered dimensions in how restrictive practices are experienced and applied.

**Discussion:**

Despite extensive national initiatives, restrictive interventions involving women have continued to rise in England, highlighting a persistent gap between policy intent and practice. The findings suggest that legislative frameworks alone are insufficient to achieve meaningful reductions without operational changes in clinical practice, organisational culture, and monitoring systems. Internationally, the study contributes rare gender-disaggregated longitudinal evidence and highlights the need for comparable monitoring systems and coordinated research to inform rights-based, trauma-informed strategies to reduce restrictive interventions in mental health services.

## 1 Introduction

Restrictive interventions, including physical restraint, seclusion, chemical restraint, and segregation, remain widespread in mental health settings internationally (1, 2). The terminology used to describe these practices varies across the literature, with terms such as ‘coercive measures’, ‘coercion’, ‘restrictive practices’, and ‘restrictive interventions’ often used interchangeably (3, 4). In the UK policy and legislative context, the term ‘restrictive interventions’ is typically used to refer to specific practices such as physical restraint, seclusion, segregation, and rapid tranquillisation (5). In this paper, we adopt the term ‘restrictive interventions’ to maintain consistency with UK policy frameworks, while recognising that the broader international literature may refer to these practices collectively as forms of ‘coercion’.

Although these practices are often justified as measures of last resort to manage safety crises, they carry substantial risks of psychological and physical harm. Women, in particular, frequently describe such experiences as traumatic and disempowering, with long-term consequences for trust, recovery, and engagement with services (6–8). Despite a decade of policy commitments to eliminate or minimise restrictive interventions in England, including Positive and Safe (5), the Women’s Mental Health Taskforce (9), and the Restrictive Practice Reduction Programme (10), quantitative evidence on national trends in restrictive interventions among women remains scarce.

Research highlights wide variation in prevalence across populations and settings, with meta-analytic estimates suggesting physical restraint occurs in approximately 14-15% of psychiatric inpatients, seclusion in 16%, and chemical restraint in up to 25% (1, 11). These rates are influenced by reporting definitions, data quality, and organisational culture (12). For people with intellectual disabilities or autism, restrictive interventions are used more frequently and often for longer durations than among other inpatients (2, 13). Yet, the extent to which these practices disproportionately affect women across diagnostic and service groups in England (and internationally) remains unknown.

The issue is not merely one of clinical management but of gendered health inequality and human rights. Restrictive interventions disproportionately affect women who have experienced interpersonal trauma or sexual violence; groups over-represented in inpatient settings (14). Such interventions can re-enact past trauma, contravening principles of trauma-informed care and equality duties under the Equality Act 2010 and the UN Convention on the Rights of Persons with Disabilities (15; Articles 14 and 25). Moreover, international reviews identify persistent under-reporting and weak transparency mechanisms (16, 17), impeding accountability and obscuring the gendered dimensions of restrictive interventions use.

Recent policy developments, notably the 18, have sought to strengthen oversight by mandating the recording and publication of restraint data. However, the real-world impact of this legislation on trends in restrictive practice remains uncertain, particularly for women. Understanding whether legislative and policy initiatives have altered practice is critical for assessing progress toward safer, rights-based mental health care.

The present study provides the first longitudinal national analysis of restrictive interventions involving women across NHS-funded mental health settings, including services for people with mental health problems, learning/intellectual disabilities and autistic people in England between 2017 and 2025. Using publicly available administrative data derived from the Mental Health Services Data Set (MHSDS) and associated Mental Health Bulletin (MHB), this study examines: (i) temporal trends in restrictive interventions involving women; (ii) age-specific and restrictive intervention type-specific trends; and (iii) the effect of the implementation of Seni’s Law on reporting patterns.

By situating these analyses within a gendered and intersectional framework, this study contributes to the evidence base for gender-sensitive policy and practice, aligning with national priorities to reduce restrictive interventions and address health inequalities among women with mental health conditions, learning disabilities, or autism.

## 2 Methods

### 2.1 Design and materials

We performed a secondary analysis using annual data from publicly available national datasets, i.e., the Mental Health Services Data Set (MHSDS), which is the primary administrative data collection covering National Health Service (NHS) funded services for people with mental health conditions, learning disabilities, and autism in England. The MHSDS provides routinely collected, anonymised service-level information submitted by healthcare providers. All data analysed in this study were obtained from official reports and open-access data files published by NHS England, which utilise and summarise MHSDS submissions. These sources provide comprehensive, quality-assured statistics that enable reliable and policy-relevant analysis of temporal patterns in service use and restrictive interventions. These outputs undergo established NHS England validation processes and quality-assurance checks, ensuring that the underlying figures are accurate, consistent, and fit for analytical and policy purposes. As a result, the dataset provides a comprehensive and methodologically robust foundation for examining temporal trends in service activity and the use of restrictive interventions.

Specifically, this investigation used the following publicly available data products:

Mental Health Bulletin (MHB): Annual national publication presenting statistics on people in contact with secondary mental health, learning disability, and autism services in England, including counts of restrictive interventions and detentions under the Mental Health Act.

KH03 (Bed Availability and Occupancy Data): Quarterly and annual datasets reporting the number of available and occupied beds in NHS organisations, used to contextualise restrictive intervention rates relative to service capacity.

Mental Health Statistics (Annual Publications): Supplementary datasets providing detailed counts of detentions, service contacts, and restrictive interventions, supporting validation and cross-referencing of trends observed in the MHB.

**Table 1** provides an overview of these publicly available data sources, including the publisher, reporting frequency, coverage, and direct web access locations from which the data were obtained.

**Table 1:** Summary of publicly available data sources used in the present investigation.

Variable	Dataset
Number of women subjected to any type of restrictive intervention	Mental Health Bulletin, Annual report, Data files
Number of women subjected to a restrictive intervention by type	Mental Health Bulletin, Annual report, Data files
Number of women subjected to a restrictive intervention by type, each region*	Mental Health Bulletin, Annual report, Data files
Number of women subjected to any type of restrictive intervention, by age.	Mental Health Bulletin, Annual report, Data files
Number of hospital beds per region	KH03 (overnight), Quarter 4, 2024-2025
Number of women detained under the Mental Health Act	Mental Health Act Statistics, Annual Figures, Data Files
Number of admissions (women only)	Mental Health Bulletin, Annual report, Data files

*This dataset contains publicly available data on the absolute number of women subjected to any type of restrictive intervention, reported per provider. Each provider was assigned to its corresponding region, and the totals were then aggregated to produce a regional sum. However, national providers (such as the Active Care Group and Priory Group Limited) were excluded from the analysis exploring regional differences, as these may not be specific to one region.

### 2.2 Participants

Only data collected for service users who identified as ‘female’ were included in this analysis to examine trends in restrictive interventions amongst women. As individual-level records are not publicly available, further demographic detail could not be examined. In the MHSDS Person Stated Gender Code records ‘gender’ as self-defined by the service user, where possible; the code “2” denotes ‘female’ and includes ‘trans women’. Where self-declaration is not possible, gender may be inferred by observation. Although the coding for ‘female’ has remained stable since 2017, changes in data collection priorities, recording practices, and the introduction of newer gender identity fields may limit comparability of this category over time.

### 2.3 Ethics

Ethical approval was not required for this investigation because the research was based solely on secondary analysis of publicly available datasets that contain no identifiable personal information.

### 2.4 Statistical analysis

Annual data reported in the data files associated with the MHSDS from 2017 to 2025 is used to provide a national-level overview of the prevalence of restrictive interventions among women accessing services for people with mental health, learning disability, and autism. Monthly data is available but prone to missing or incomplete records, which could introduce bias and obscure true patterns (19). Aggregating the data annually reduces the influence of such gaps and random short-term fluctuations, allowing for a clearer assessment of long-term trends (20). Furthermore, policy decisions and resource planning are typically informed by yearly trends rather than monthly variation, making annual data more directly actionable (21, 22). Therefore, while monthly trends may exist, the use of annual data ensures a conservative and reliable basis for guiding policy recommendations.

All data analysis was performed using R (23) with model diagnostics performed using standard R packages. As this study uses publicly available aggregated annual data, records with missing or incomplete information (e.g., sex, age, intervention type) are already excluded from the published counts. Therefore, no imputation was performed. All trend analyses were interpreted in the context of dataset coverage.

Given the aggregated nature of the available data, individual-level descriptive statistics were not possible. Instead, we summarised the total number (henceforth referred to as ‘absolute counts’) of women subjected to restrictive interventions; the absolute counts of women involuntarily detained under the Mental Health Act (24); and, calculated the rate of women subjected to restrictive interventions per 1,000 of those detained. Additionally, cross-sectional regional visualisations (e.g., infographics) were produced to illustrate the absolute counts of women exposed to any restrictive intervention. To provide a contextualised view of restrictive practice across different regions, these visualisations incorporated the total number of beds within each region to account for variations in service capacity (24).

For longitudinal analysis, we examined temporal trends in the use of restrictive interventions among women accessing NHS-funded secondary mental health services, including for those with a learning disability or autism. Using publicly available data, regression modelling was employed to quantify changes over time. Time-series regression techniques such as Poisson or Negative Binomial regression for count data (overdispersion was assessed to guide model choice) were performed. All models model the absolute counts of the outcome of interest while accounting for the number of women detained each year (24), effectively modelling the rate of restrictive interventions per detained woman. Year was treated as a continuous predictor for all models. Specifically, we examined:

#### 2.4.1 Trends of restrictive interventions

- Overall temporal trends in restrictive interventions: We modelled the absolute counts of women subjected to restrictive interventions from 2017 to 2025 whilst accounting for those detained.
- Impact of the Mental Health Units (Use of Force) Act 2018 (‘Seni’s Law’): We extended the previously outlined regression model to include the implementation of Seni’s Law as of 2021. Implementation of Seni’s Law was treated as a categorical predictor to estimate annual changes in reporting of restrictive intervention rates, and we examined both the main effects (with year as a significant predictor) and the interaction effect between the implementation and year.

#### 2.4.2 Trends of restrictive intervention by age group

We modelled the absolute counts of women subjected to restrictive interventions (while accounting for those detained) from 2017 to 2025 for each age group: ‘under 18’, ‘18 to 24’, ‘25 to 29’, ‘30 to 39’, ‘40 to 49’, ‘50 to 59’ and ‘60 and over’. Due to changes in reporting age groups, ‘under 18’, ‘18 to 24’ and ‘25 to 29’ could only be reported from data available in the 2019–2020 annual reports. Separate models were performed as outcomes may not be mutually exclusive. Also, due to the nature of the data, interaction effects could not be estimated.

#### 2.4.3 Temporal trends by type of restrictive intervention

We modelled the absolute counts of women subjected to restrictive interventions (while accounting for those detained) from 2017 to 2025 for each type of intervention: Physical restraint (Prone only), Chemical restraint (rapid tranquilisation only), Mechanical restraint, Seclusion, and Segregation. Separate models were performed as outcomes may not be mutually exclusive. **Supplementary Material** provides the definition of the type of restrictive intervention as defined by the MHSDS. Due to changes in the reporting of types of restrictive interventions, data for chemical restraint (rapid tranquilisation only) for years 2017–2018 and 2018–2019 were excluded from analysis.

To check the sensitivity of the results, all models were re-estimated using the number of women admissions to services, rather than the number of women detained each year (see **Supplementary Material**). Additionally, all analyses were repeated using only service users who identified as male (see **Supplementary Material**) to explore whether the findings were gender specific.

## 3 Results

### 3.1 Descriptive findings

**Table 2** provides the absolute counts of women subjected to restrictive intervention and women detained under the Mental Health Act as well as the rate of restrictive intervention per 1,000 women detained between 2017 to 2025. This demonstrates that the rate of restrictive interventions for women detained under the mental Health Act (including for per 1,000 women detained) has increased from 2017 to 2025.

**Table 2:** Summary of reported restrictive interventions (RI) experienced by women between 2017 to 2025.

Year	Number of admissions (Women only)	Number of women detained under the Mental Health Act	Restrictive interventions	
			Number of women subjected to RI	Rate of RI per 1,000 detained
17-18	57,325	22,753	4,246	186.61
18-19	58,861	23,543	4,450	189.02
19-20	58,339	24,007	5,448	226.93
20-21	53,092	25,122	6,047	240.71
21-22	52,958	24,693	6,268	253.84
22-23	47,657	22,928	7,405	322.97
23-24	48,779	24,407	7,854	321.79
24-25	47,402	23,906	8,877	371.33

**Figure 1:**
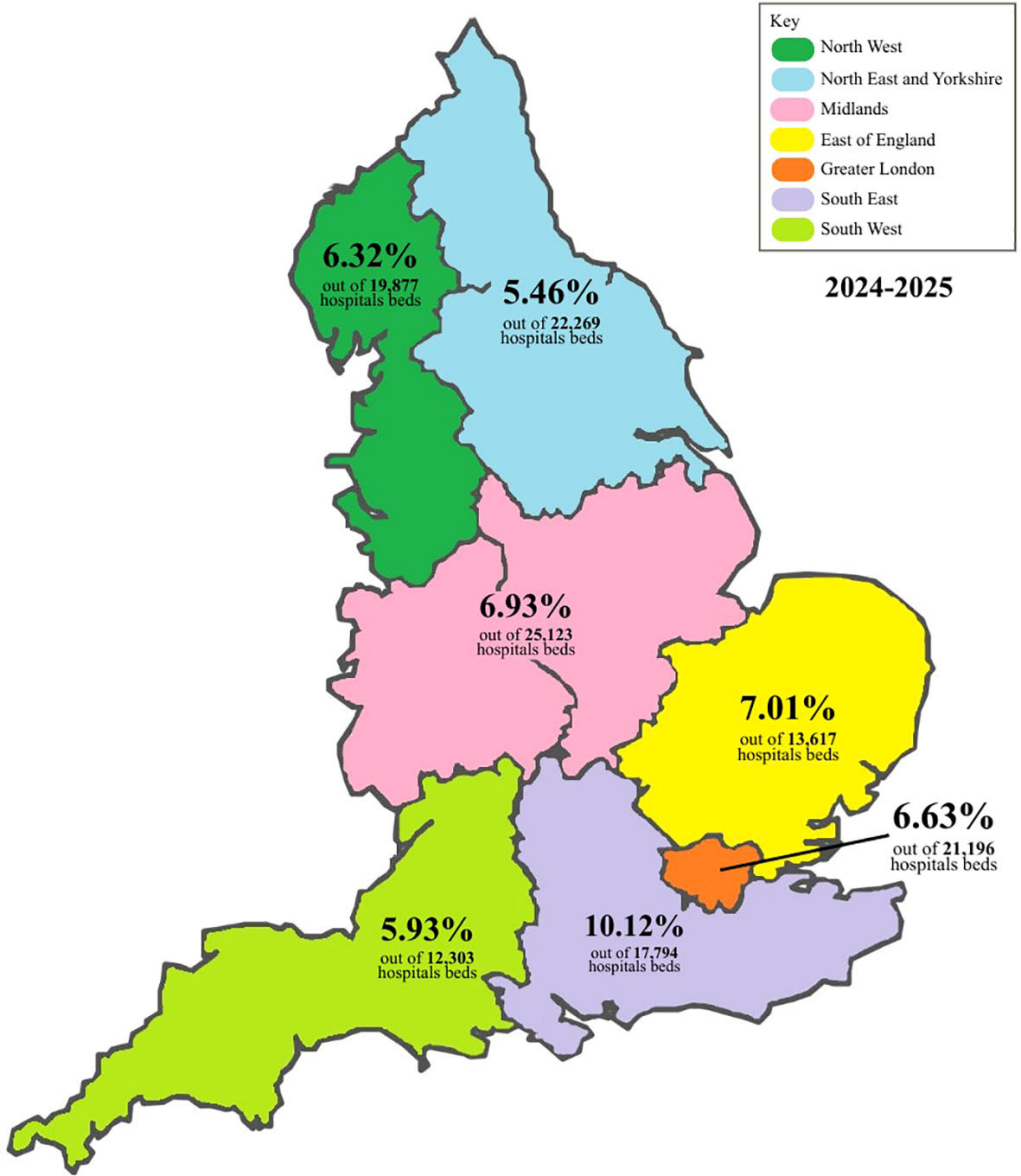
Proportion of women subjected to any restrictive intervention relative to the number of hospital beds across English regions (2024–2025).

**Figure 1** provides descriptives of the proportion of women subjected to any restrictive intervention relative to the number of hospitals beds across multiple regions of England. The figure illustrates that most regions cluster around 6-7%, including the North-West, North-East and Yorkshire, Midlands, South-East, and Greater London. The East of England stands out with the highest proportion at 10.12%, indicating a markedly greater allocation relative to other areas. In contrast, the South-West has the lowest proportion at 5.46%, despite having one of the largest total bed capacities. Overall, the pattern suggests that regional differences in service provision, demand, or bed categorisation contribute to the uneven distribution across the country. Therefore, it suggests that where a woman is treated appears to influence her likelihood of being subjected to restrictive interventions.

### 3.2 Longitudinal analysis

For all the models estimated, **Supplementary Material** presents all the regression results, dispersion diagnostics, and comparative model fit statistics (AIC) for the Poisson and negative binomial models. Sensitivity analysis (see **Supplementary Material**) demonstrates that the observed temporal trends are robust to the choice of denominator and not substantially influenced by changes in the size of the admitted or detained populations.

#### 3.2.1 Trends of restrictive interventions 2017-2025

Restrictive interventions involving women increased by an estimated 11% per year (IRR = 1.107, 95% CI [1.09, 1.12]). This finding suggests that, after accounting for the number of women detained each year, the number of women subjected to any form of restrictive intervention has increased steadily since 2017. Analysis was performed again for service users identifying as male (see **Supplementary Material***)* and descriptive comparisons demonstrate similar trends to females; this suggests the results are not likely gender specific.

Further analysis was performed to examine whether the implementation of Seni’s Law (in 2021) affected the trend in reporting restrictive interventions. However, there was no significant difference in trends before and after 2021, indicating a no change since the implementation of Seni’s law.

#### 3.2.2 Trends of restrictive intervention by age group

Regressions were performed to examine trends in restrictive interventions for each of the age groups between 2017 and 2025 (see **Supplementary Material**). To restate, changes reporting in how age categories were reported in the 2017–2018 and 2018–2019 datasets meant that data for the following age groups could not be analysed and therefore, were excluded: ‘under 18’, ‘18 to 24’, and ‘25 to 29’.

For women under 18 years of age, rates of restrictive interventions decreased by an estimated 14% per year (IRR = 0.86, 95% CI [0.76, 0.96]). This finding suggests that, after accounting for the number of women detained each year, women under 18 who were subjected to any form of restrictive intervention has decreased steadily since 2017.

By contrast, for women over the age of 18 there was a steady increase in the use of restrictive intervention since 2017 per year (ranging between 9.5 to 12%). For women aged between 18 and 24, rates of restrictive intervention increased by 9.5% per year (IRR = 1.095, 95% CI [1.06, 1.13]); for women aged between 25 to 29, this was estimated 12% per year (IRR = 1.12, 95% CI [1.10, 1.14]); for women aged 30 to 39, this was estimated 11% per year (IRR = 1.11, 95% CI [1.10, 1.13]); for women aged between 40 to 49, this was estimated 12% per year (IRR = 1.12, 95% CI [1.10, 1.13]); for women aged between 50 to 59, this was estimated 10% per year (IRR = 1.10, 95% CI [1.09, 1.12]); and, for women aged 60 and over, this was estimated 11% per year (IRR = 1.11, 95% CI [1.10, 1.12]).

Collectively, the findings indicate a consistent increase in the proportion of women aged 18 and over subjected to any form of restrictive intervention, relative to the total number detained, between 2017 (or 2019) and 2025. In contrast, the number of females under 18 experiencing such interventions decline between 2019 and 2025.

**Figure 2:**
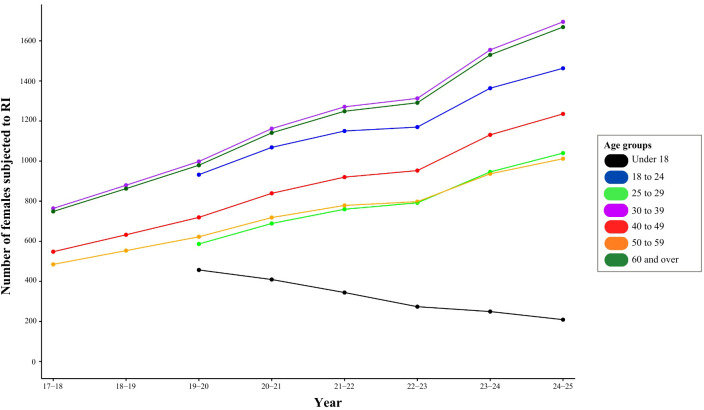
Trends in restrictive interventions (RI) across age groups.

**Figure 2** provides an overview of the trends in the use of restrictive interventions across different age groups, relative to the number of women detained under the Mental Health Act each year. This shows that restrictive interventions among women have declined only in those under 18 (14% decrease), while all adult age groups experienced increases of around 10–12% between 2017–18 and 2024-25. This indicates a divergence in trends, with reductions for minors but steady growth in RI use among adults; possibly suggesting differential policy effectiveness across services for minors and adults.

Analysis was performed again for service users identifying as male (see **Supplementary Material***)* and trends appear to be comparable to that observed in the previously presented figure for females. However, when comparing across figures, the graph could suggest that for males under 18 the rate of restrictive intervention remains stable rather than decreasing.

#### 3.2.3 Temporal trends by type of restrictive intervention

Regressions were performed to examine trends for type of restrictive intervention between 2017 and 2025. Due to changes in the reporting of types of restrictive categories, data for chemical restraint (rapid tranquilisation only) years 2017–2018 and 2018–2019 were excluded from analysis.

There was a steady increase in women who are subjected to chemical restraint (rapid tranquilisation only), seclusion and segregation 2017-2025. Rates of chemical restraint involving women increased by an estimated 8% per year (IRR = 1.08, 95% CI [1.03, 1.11]). Rates of seclusion involving women increased by an estimated 5.3% per year (IRR = 1.053, 95% CI [0.04, 1.05]). Rates of segregation involving women increased by an estimated 14% per year (IRR = 1.14, 95% CI [1.03, 1.25]). In contrast, there was no significant increase or decrease for the use of physical (prone only) and mechanical restraint.

Together, the findings indicate a consistent increase in the proportion of women subjected to chemical restraint (rapid tranquilisation only), seclusion and segregation, relative to the total number detained, between 2017 and 2025. However, the number of women subjected to physical (prone only) and mechanical restraint did not change; suggesting that the rates of these remained stable between 2017 and 2025.

**Figure 3:**
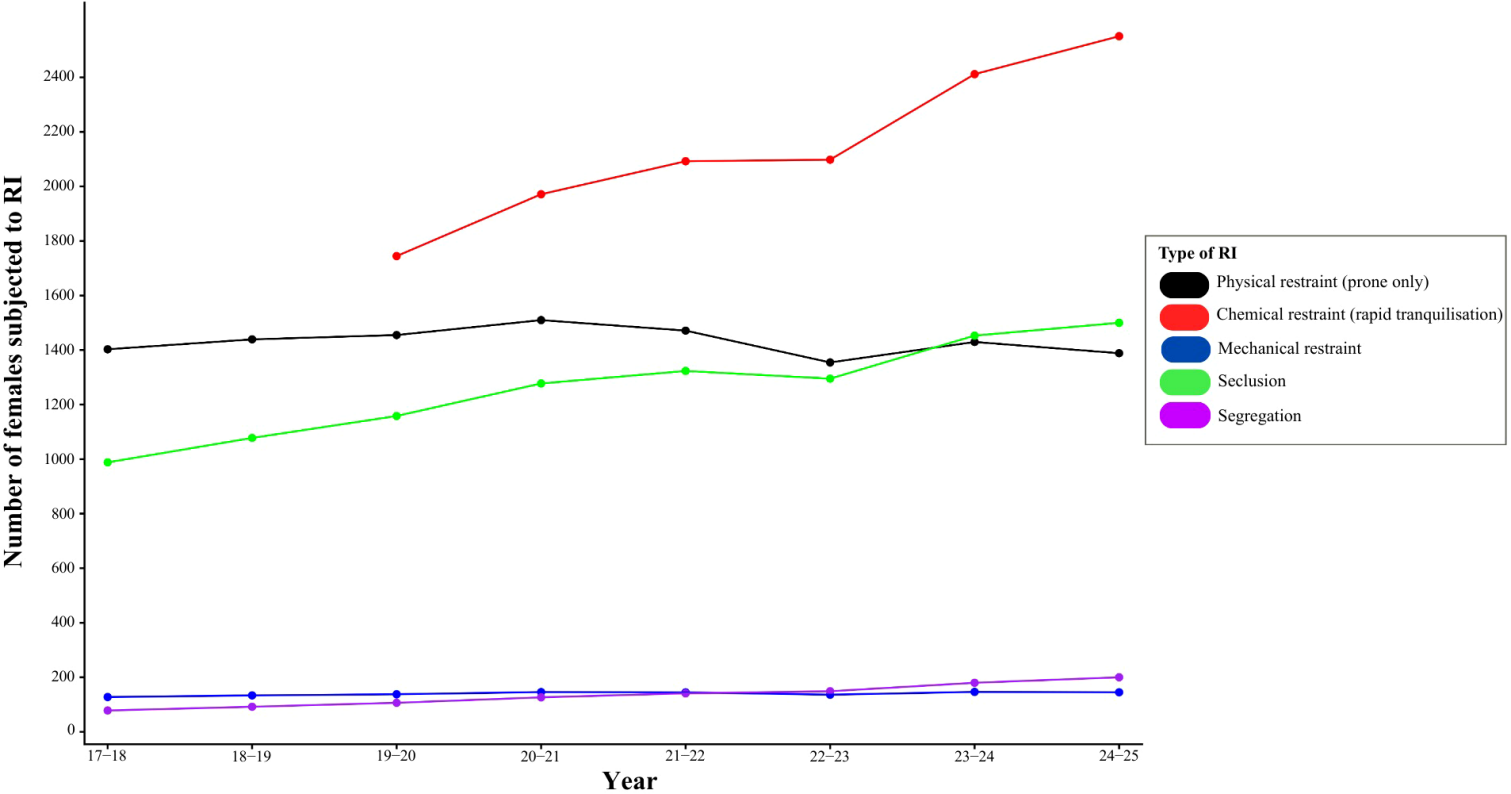
Trends in types of restrictive interventions (RI), 2017–2025.

Supplementary analysis also provides additional results for available trends for alternative types of chemical restraint (non-rapid tranquilisation, oral and other). **Figure 3** illustrates the trends in different types of restrictive interventions from 2017 to 2025, relative to the number of women detained under the Mental Health Act each year. The figure highlights changes in the use and frequency of each intervention type over time, providing a clear visual comparison of how the prevalence of each restrictive intervention has evolved throughout the observed period. The figure illustrates continued use of all types of restrictive interventions for women from 2017–18 to 2024–25, with several forms showing gradual increases over time.

Analysis was performed again for service users identifying as male (see **Supplementary Material***)* and observational comparisons suggest a gender difference in the use of specific interventions. Rapid tranquillisation (chemical restraint) appears to be used more frequently than seclusion among females, whereas the opposite pattern is observed for males.

## 4 Discussion

### 4.1 Summary of the findings

This study provides the first national longitudinal analysis of restrictive interventions involving women across NHS-funded mental health services in England. Despite sustained policy and legislative efforts to promote least-restrictive and trauma-informed care (5, 9, 10), the findings demonstrate a consistent increase in the rate of restrictive interventions involving women between 2017 and 2025. This upward trend persisted after accounting for changes in detention rates, indicating a substantive rise in the use of restrictive interventions rather than an artefact of service throughput or population size. Increases were particularly pronounced for chemical restraint, seclusion, and segregation, while physical and mechanical restraint remained largely stable. No measurable change in trends was observed following implementation of the 18.

Taken together, these findings challenge assumptions that restrictive interventions are declining in response to national policy initiatives. Instead, they suggest that for adult women, restrictive interventions remain embedded within routine care practices, echoing concerns raised in previous national analyses of restraint reporting trends (24) and international comparative work (12) and raising important questions about the effectiveness of current reduction strategies and the conditions under which policy reforms translate into practice.

### 4.2 Interpretation and service-level implications

The observed increase in chemical restraint, seclusion, and segregation aligns with evidence suggesting that restrictive interventions may shift form rather than diminish in overall use (12) - some form of displacement. While reductions in physical restraint have been reported in some settings following targeted training and restraint-reduction initiatives (10), these gains may be offset by increased reliance on pharmacological containment or spatial separation. Similar substitution effects have been documented internationally, particularly in contexts of staffing shortages and high clinical acuity (8, 25).

The observed pattern may therefore reflect substitution between different forms of restrictive practice rather than an overall reduction. While reductions in physical restraint are often interpreted as evidence of progress in restrictive-practice reduction programmes, increases in chemical restraint, seclusion, or segregation may indicate a shift towards alternative mechanisms of containment rather than a transition to genuinely less restrictive care. Evidence from international research suggests that pharmacological restraint, seclusion, and segregation can carry substantial psychological and physical risks, including sedation-related adverse events, loss of autonomy, and re-traumatisation, particularly for individuals with prior experiences of violence or institutionalisation (8, 12, 16).

Qualitative studies also highlight that service users frequently perceive these practices as similarly restrictive, regardless of the specific form they take (6). From this perspective, the observed pattern may represent partial progress in reducing the most physically dangerous forms of restraint, but not necessarily a reduction in reliance on restrictive interventions overall. Instead, it may indicate a reconfiguration of risk management strategies within mental health services, where different forms of restriction are used interchangeably in response to organisational pressures such as staffing levels, ward environments, and perceptions of safety (8, 25).

These patterns must also be interpreted within the wider service context of increasing demand, workforce instability, and post-pandemic pressures on inpatient care. Qualitative studies consistently highlight how organisational cultures of risk aversion, moral distress, and limited staffing capacity shape the use of restrictive interventions (16, 25). From a systems perspective, the findings underscore the need to examine restrictive interventions not solely as individual clinical decisions, but as outcomes of structural and organisational conditions.

The decline of restrictive practice use among women under 18 contrasts with rising trends in adult groups, possibly reflecting targeted investment in child and adolescent trauma-informed care (e.g., NHS CAMHS Quality Improvement programmes: 26, 27). This divergence underscores the importance of age-specific policy interventions.

### 4.3 Gendered inequalities, stigma and intersectionality

The persistent rise in restrictive interventions among adult women raises significant concerns from both equality and safeguarding perspectives. Women in inpatient and secure settings are disproportionately likely to have histories of trauma, abuse, restrictive interventions risk re-traumatisation and long-term disengagement from care (6, 14). From a health inequalities perspective, restrictive interventions may function as mechanisms through which structural disadvantage and stigma are reproduced within mental health systems.

Although intersectional analyses were not possible within the constraints of aggregated administrative data, existing research indicates that restrictive interventions are more prevalent among racialised women and women with learning disabilities or autism (2, 13). International reviews further highlight how the absence of routinely collected, intersectional data limits accountability and obscures inequalities in the use of restrictive interventions (12). Addressing these data gaps is therefore essential to advancing gender-equitable and rights-based care. In the English context, future linkage of MHSDS with demographic identifiers is essential to monitor these compounded risks.

### 4.4 Legislative learning and limits of statutory reform

The absence of a detectable change following implementation of *Seni’s Law* highlights the limitations of legislation as a standalone mechanism for reducing restrictive interventions. While *Seni’s Law* strengthened requirements for recording, oversight, and transparency, evidence from England and elsewhere suggests that statutory reporting alone is insufficient to disrupt entrenched organisational cultures or clinical risk-management practices (16, 17). This finding should not be interpreted as legislative failure, but rather as evidence that legal frameworks must be accompanied by sustained investment in workforce training, reflective supervision, and organisational culture change. Without parallel efforts to address fear-based (also called ‘closed’) cultures, moral distress, and resource constraints, transparency requirements may improve visibility without achieving meaningful reductions in restrictive practice (12).

### 4.5 Strengths and limitations

This study’s strengths include its national coverage, use of quality-assured administrative data, and longitudinal design spanning periods of major policy and legislative change. The inclusion of sensitivity analyses further strengthens confidence in the robustness of observed trends (21, 22).

However, limitations must be acknowledged. Annual aggregation limits insight into within-year variation and short-term responses to policy or service changes (20). Variations in reporting definitions across years may introduce minor discontinuities, and under-reporting remains an inherent limitation of administrative datasets (28). The use of aggregated data also precluded individual-level or intersectional analyses, constraining examination of differential impacts across ethnic, diagnostic, or socioeconomic groups. Additionally, although attempts were made to compare trends in restrictive interventions across genders (service users identified as female and male), individual-level data would also be required to conduct more robust inferential statistical analyses to confirm gender differences. Further analysis to confirm gender differences is imperative as visual comparisons of trends in large, aggregated datasets may mask subtle yet significant differences that may not be reliably detected without more robust statistical analysis. Lastly, although the number of women subjected to restrictive interventions was controlled for by the number of women detained, it cannot be assumed that the reported figures represent unique individuals. Some women may have experienced restrictive interventions across multiple locations within a single year, potentially leading to duplicate entries within the aggregated data. Despite these limitations, this study provides the most comprehensive national overview of restrictive interventions involving women in England to date.

A further limitation is that more extensive international comparison was not possible because the wider literature contains very little gender-disaggregated longitudinal trend data and uses inconsistent definitions and reporting practices, limiting direct comparison across jurisdictions.

### 4.6 International relevance and future research

A more detailed international comparison is constrained by the current state of the evidence base. International studies consistently show that restrictive interventions remain common in mental health settings, but rates vary substantially across countries and services because of differences in definitions, measurement, reporting systems, and organisational culture (1, 12). Reviews also show that women experience restrictive interventions as particularly harmful, often retraumatising, and closely tied to gendered power, violence, and loss of autonomy (6–8). However, most international studies either combine men and women, focus primarily on qualitative experiences, or report prevalence without providing gender-disaggregated longitudinal trend data (2, 12). As a result, direct quantitative comparison with the present findings is limited.

Within those constraints, our results are broadly consistent with the international literature in showing persistent reliance on restrictive interventions despite policy commitments to least-restrictive care (12). They extend that literature by providing rare national longitudinal evidence showing rising rates among adult women, stability in some intervention types but increases in chemical restraint, seclusion, and segregation, and no detectable change following legislative reform. In this sense, the present study contributes not only national evidence from England but also a missing gender-focused benchmark for future cross-national comparison.

In light of this, future research should prioritise linkage of administrative, regulatory, and safeguarding datasets to support intersectional analyses and longitudinal evaluation of policy interventions. Collaborative research using harmonised administrative and qualitative data would support cross-national learning on effective reduction strategies and strengthen understanding of how human rights frameworks, such as the UN Convention on the Rights of Persons with Disabilities, can be operationalised in practice. Building this comparative evidence base is essential for advancing global accountability and promoting safer, rights-based, and gender-equitable mental health care. Mixed-methods approaches integrating quantitative monitoring with women’s lived experiences are essential for understanding not only how often restrictive interventions occur, but how they are experienced, justified, and resisted within different care contexts (7, 8).

### 4.7 Policy implications

While this paper highlights that overall restrictive practice trends in England are similarly increasing for both men and women, their impact remains a gendered issue. What is important to note, regardless of any similarities or differences, is that legislative compliance alone is insufficient to reduce restrictive interventions; it must be accompanied by sustained cultural reform, adequate staffing, and transparent, intersectional data monitoring. Addressing restrictive interventions involving women therefore requires coordinated action across organisational, commissioning, and national policy levels.

Reducing restrictive interventions requires not only legislative frameworks, but also their translation into concrete operational practices within mental health services. Evidence from international quality-improvement initiatives suggests that meaningful reductions in restrictive interventions are typically associated with organisational systems that embed routine monitoring, leadership accountability, and structured learning from incidents. These systems may include regular multidisciplinary review of restrictive interventions, the use of individualised crisis or calming plans developed with service users, transparent monitoring of restrictive-practice data at ward and organisational levels, and structured debriefing processes involving both staff and service users following restrictive events. Workforce development, including training in de-escalation, trauma-informed care, and person-centred communication, is also central to reducing reliance all types of restrictive interventions. Such operational practices help translate broad legislative and policy commitments into day-to-day clinical decision-making and organisational culture change.

Importantly, these operational strategies should also incorporate gender-sensitive and trauma-informed approaches. Women in inpatient settings are disproportionately likely to have histories of violence, abuse, and coercion, and restrictive interventions may therefore carry heightened risks of re-traumatisation and disengagement from care (6, 14). Ensuring that restrictive-practice reduction strategies account for these gendered experiences is essential for advancing equitable and rights-based mental health care.

**Table 3** summarises priority recommendations derived directly from the study’s findings and aligned with existing regulatory and policy frameworks, highlighting how national commitments to reduce restrictive interventions can be operationalised through concrete organisational practices.

**Table 3:** Policy and practice recommendations to reduce restrictive interventions involving women in mental health settings.

Level	Priority recommendation	Rationale informed by findings and evidence
Organisational (provider/NHS trust level)	Embed gender-sensitive, trauma-informed training across multidisciplinary teams, with explicit focus on women’s experiences of restrictive interventions and re-traumatisation.	Rising rates of restrictive interventions among adult women, alongside qualitative evidence of harm and stigma, indicate that current practice does not sufficiently account for gendered trauma histories (6, 7).
	Mandate structured post-incident debriefing and reflective supervision after each restrictive event for both staff and service users following any restrictive intervention.	Persistent use of restrictive practices is associated with fear-based organisational cultures and moral distress among staff; reflective supervision is a recognised mechanism for reducing repeat use (16, 25).
	Integrate restrictive-intervention metrics into routine patient-safety and equality dashboards at ward and trust level (linking restraint data with patient demographics).	The study demonstrates sustained increases in restrictive interventions despite policy commitments, highlighting the need for routine, transparent monitoring linked to governance structures (24).
Commissioning and regulation	Require standardised, gender-disaggregated reporting of restrictive interventions through NHS Standard Contracts and Integrated Care Board performance frameworks.	Aggregated national data obscure intersectional inequalities; consistent reporting is essential for accountability and compliance with equality duties (12).
	Align Care Quality Commission inspection frameworks with indicators of restrictive-practice reduction and closed-culture prevention.	Ongoing use of segregation and seclusion reflects organisational risk cultures identified in regulatory reviews of closed settings (17).
National policy	Undertake post-legislative evaluation of the Mental Health Units (Use of Force) Act 2018 (*Seni’s Law*), focusing on implementation fidelity and organisational impact rather than reporting compliance alone.	The absence of a detectable change following implementation suggests that legislation alone is insufficient to alter entrenched practices without cultural and resource support.
	Integrate restrictive-practice indicators into national women’s mental health and patient-safety strategies.	Divergent trends by age group indicate that targeted policy attention can reduce restrictive practice; adult women currently lack equivalent strategic focus.
	Invest in development of intersectional administrative datasets and lived-experience-led monitoring frameworks.	Lack of routinely available intersectional data limits understanding of compounded inequalities among women with learning disabilities, autism, and from racialised groups.

## 5 Conclusion

Despite sustained national commitments to reduce restrictive interventions, this study demonstrates that restrictive interventions involving women have continued to rise in England over the past eight years. Drawing on national longitudinal data, the findings highlight a persistent disconnect between policy intent and practice, particularly in adult mental health settings. These trends reaffirm the need for trauma-informed, rights-based, and transparent approaches to care. Reducing restrictive interventions and other coercive practices is not solely a matter of regulatory compliance, but of justice; requiring that women’s safety, dignity, and autonomy are placed at the centre of mental health service design, delivery, and oversight.

## Data Availability

The original contributions presented in the study are included in the article/**Supplementary Material**. Further inquiries can be directed to the corresponding author.
