# Supplementary figures and images for: A longitudinal analysis of the prevalence of restrictive interventions involving women with mental health conditions, learning disabilities or autism in mental health services in England
Source: Front Psychiatry. 2026 Apr 14;17:1787826. doi: 10.3389/fpsyt.2026.1787826 (PMC13121310; doi:10.3389/fpsyt.2026.1787826)

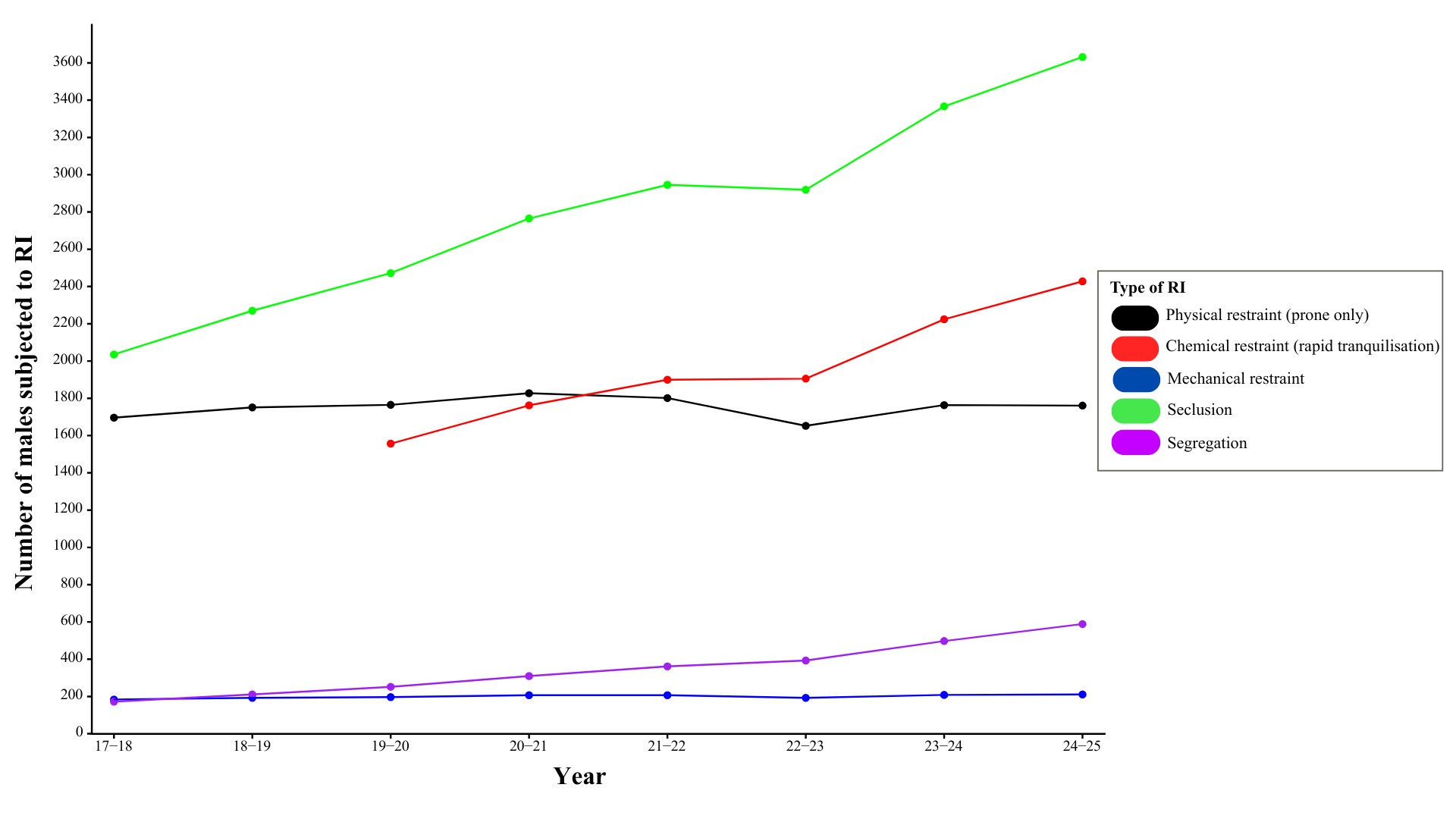

Supplement: Supplementary file 1 [file Image1.jpeg]

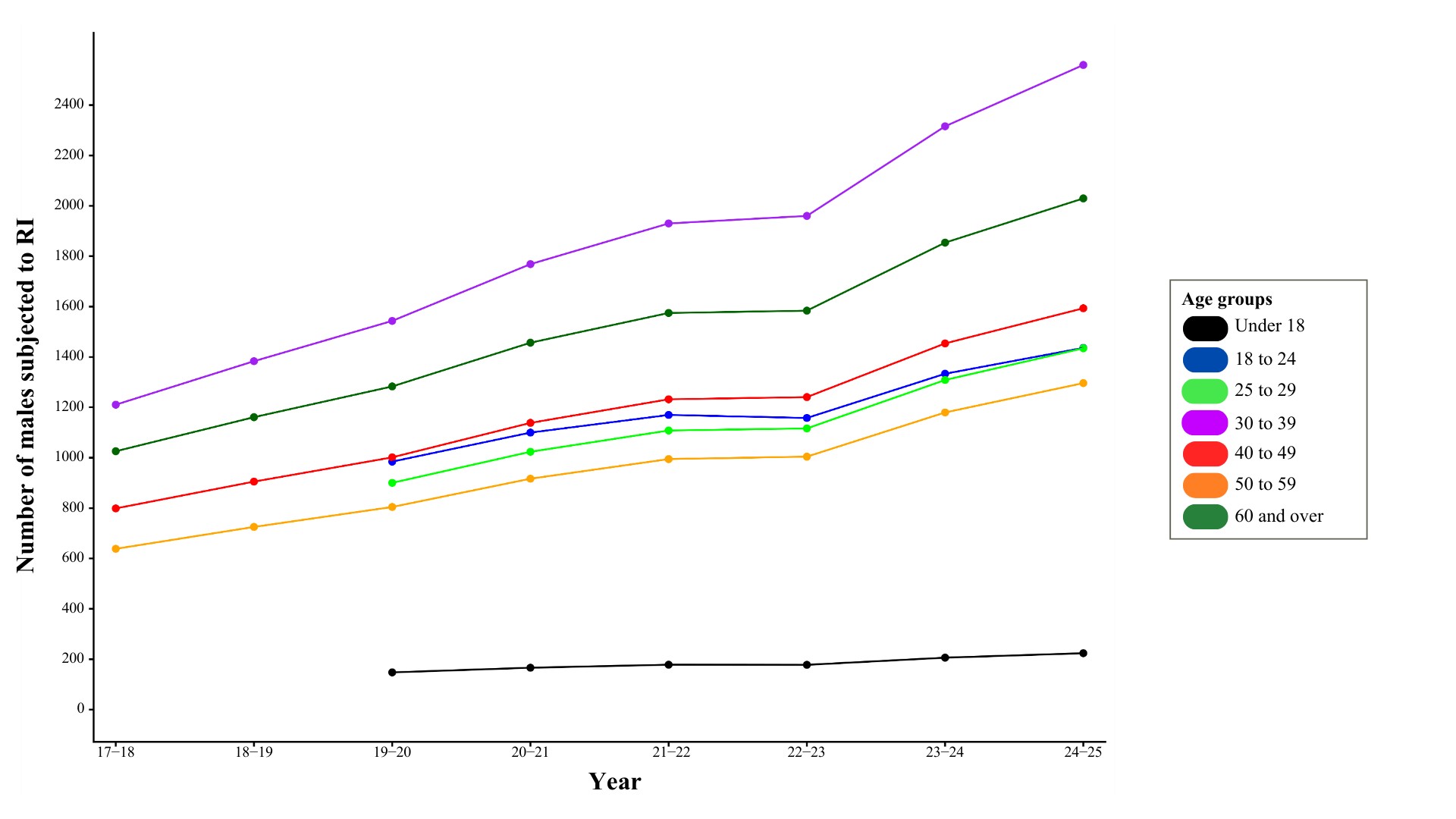

Supplement: Supplementary file 2 [file Image2.jpeg]

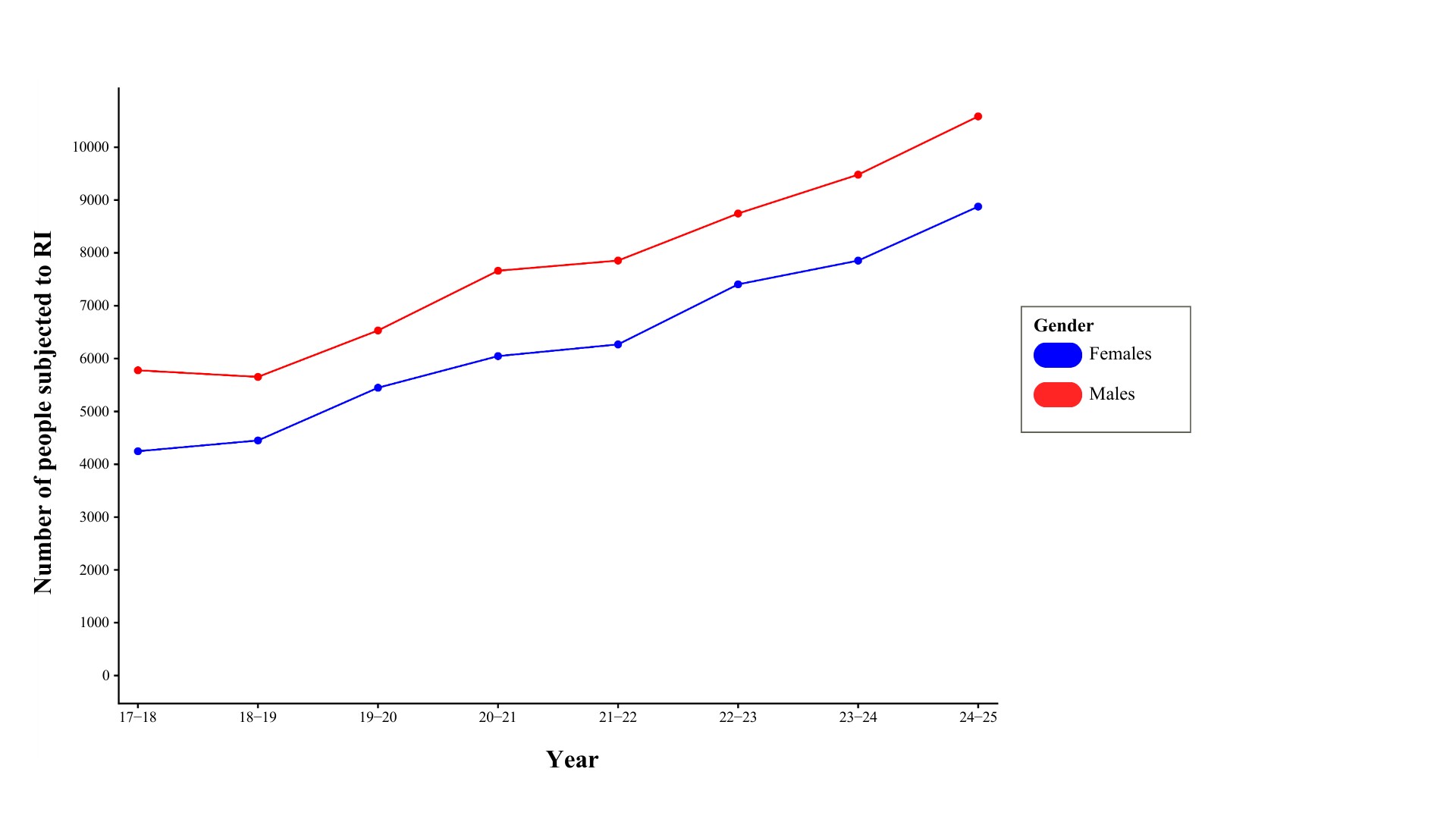

Supplement: Supplementary file 3 [file Image3.jpeg]
